# Dihydromyricetin Ameliorates Cardiac Ischemia/Reperfusion Injury through Sirt3 Activation

**DOI:** 10.1155/2019/6803943

**Published:** 2019-04-16

**Authors:** Liping Wei, Xuseng Sun, Xin Qi, Yufan Zhang, Yuanyang Li, Yue Xu

**Affiliations:** ^1^Department of Cardiology, Tianjin Union Medical Center, Nankai University Affiliated Hospital, Tianjin, China; ^2^Tianjin Medical University, Tianjin, China; ^3^Tianjin University of Traditional Chinese Medicine, Tianjin, China

## Abstract

During myocardial infarction, quickly opening the occluded coronary artery is a major method to save the ischemic myocardium. However, it also induces reperfusion injury, resulting in a poor prognosis. Alleviating the reperfusion injury improves the prognosis of the patients. Dihydromyricetin (DHM), a major component in the* Ampelopsis grossedentata*, has numerous biological functions. This study aims to clarify the effects of DHM under the ischemia/reperfusion (I/R) condition. We elucidated the role of Sirt3 in the cardiomyocyte response to DHM based on the hearts and primary cardiomyocytes. Cardiac function, mitochondrial biogenesis, and infarct areas were examined in the different groups. We performed Western blotting to detect protein expression levels after treatments. In an in vitro study, primary cardiomyocytes were treated with Hypoxia/Reoxygenation (H/R) to simulate the I/R. DHM reduced the infarct area and improved cardiac function. Furthermore, mitochondrial dysfunction was alleviated after DHM treatment. Moreover, DHM alleviated oxidative stress indicated by decreased ROS and MnSOD. However, the beneficial function of DHM was abolished after removing the Sirt3. On the other hand, the mitochondrial function was improved after DHM intervention in vitro study. Interestingly, Sirt3 downregulation inhibited the beneficial function of DHM. Therefore, the advantages of DHM are involved in the improvement of mitochondrial function and decreased oxidative stress through the upregulation of Sirt3. DHM offers a promising therapeutic avenue for better outcome in the patients with cardiac I/R injury.

## 1. Introduction

Despite progress in the treatment of myocardial infarction (MI), it still remains high mortality [1, 2]. Revascularization therapy itself can cause reperfusion injury to the myocardium in patients with MI [3]. Reopening blood flow rapidly can induce the accumulation of ROS, and the blood waste can enter the ischemic area and cause secondary damage, known as myocardial ischemia/reperfusion (MI/R) injury.

Dihydromyricetin (DHM) is the key ingredient in the traditional Chinese herb* Ampelopsis grossedentata* [4–6]. According to previous studies, DHM has numerous advantages in treating obesity, nonalcoholic fatty liver disease, atherosclerosis, and diabetic cardiomyopathy [4, 6–8]. Furthermore, DHM enhances glucose uptake, decreases the inflammatory reaction, and alleviates oxidative stress [6, 9, 10]. We explore the function and underlying mechanism of DHM against MI/R injury.

Mitochondria are cellular energy factories that supply the ATP to sustain the activity of the heart. Mitochondrial dysfunction induces an impaired energy supply resulting in heart dysfunction [11, 12]. Moreover, abnormal mitochondria are responsible for cell apoptosis and death. A phenotype of mitochondrial biogenesis dysfunction was observed during cardiac I/R injury [11, 12]. It was suggested that mitochondria take part in the pathological process of cardiac I/R injury. Illuminating the underlying mechanisms will provide novel therapeutics to improve mitochondrial function and optimize the prevention and treatment for cardiac I/R injury.

Thus far, Sirt1-7 have been identified [13, 14]. These sirtuins have distinct functions and subcellular locations. Sirt3 regulates mitochondrial biogenesis and function [15]. Recent studies have shown that Sirt3 improves aging-associated cardiac abnormalities, maintains cardiac contractile function, and attenuates the extent of fibrosis in cardiac hypertrophy [16–18]. Moreover, Sirt3 participates in the regulation of oxidative stress and cellular apoptosis [18, 19]. The ROS level indicates the extent of the oxidative damage. Sirt3 regulates manganese superoxide dismutase (MnSOD), which is a major antioxidant enzyme [20, 21]. Consistently, removing Sirt3 increases oxidative damage induced by ROS and mitochondrial dysfunction [18, 22]. Targeting Sirt3 may be a novel strategy in improving the prognosis of MI/R.

Therefore, we attempted to determine whether DHM is involved in alleviating cardiac I/R injury. Moreover, we investigated whether the protective function of DHM is associated with the improvement of mitochondrial function and decreased oxidative stress through the upregulation of Sirt3.

## 2. Methods

### 2.1. Study Approvals and Groups

The use of animals in this study conformed to the National Institutes of Health Guidelines on the Use of Laboratory Animals and was approved by the Tianjin Union Medical Center Committee on Animal Care. 8–12-week-old male mice were randomized into six groups: WT+ sham group, Sirt3^−/−^ + sham group, MI/R group, Sirt3^−/−^ + MI/R group, MI/R + DHM group, and Sirt3^−/−^ + MI/R + DHM group.

DHM (100 mg/kg/d) purchased from Sigma-Aldrich was given intragastrically for 8 weeks. Then, we constructed the MI/R model.

### 2.2. Myocardial Ischemia/Reperfusion

MI/R was performed as previously described [12]. After inhaling 2% isoflurane anesthesia, a skin cut was made over the left chest. Then, a slipknot was made on the one-third of the left anterior descending artery. After 30 min, we reopened the chest to loosen the slipknot.

### 2.3. Echocardiography

The cardiac function of mice was examined as previously described [12]. The experimenters were blind to group assignment and outcome assessment.

### 2.4. Transmission Electron Microscopy (TEM)

TEM was used to examine the mitochondrial ultrastructure. Mouse hearts were quickly rinsed in phosphate buffer saline (PBS). The primary cardiomyocytes were collected using trypsin. The samples were examined as previously described [23].

### 2.5. Cardiomyocyte Apoptosis

Caspase-3 activity was detected as previously described [24].

### 2.6. Assessment of Cardiomyocyte Injury

The ischemic tissues release lactate dehydrogenase (LDH) and creatine kinase-MB (CK-MB) to the arterial blood. LDH and CK-MB activity were detected spectrophotometrically with a commercially available assay.

### 2.7. Cardiomyocytes Culture

The ventricular cardiomyocytes were isolated from 1-day-old mice as previously described [23]. We used the DMEM supplemented with 10% fetal bovine serum (HyClone) to prepare the medium. We enriched cardiomyocytes by removing fibroblasts and plated cardiomyocytes in gelatin-coated plates.

### 2.8. Adenoviruses Transfection

We used the adenoviruses (Ad-sh-Sirt3, 1 × 10^9^ TU / ml) to knockdown the Sirt3 and the value of MOI was 100. The adenoviruses were transduced 24 h after DHM (1 *μ*M) treatment. After 36 h, we constructed the Hypoxia/Reoxygenation (H/R) model. The cells were cultured with the Hypoxia environment for 4 h (simulated ischemia for 4 h) and then cultured with the normal environment for 4 h (simulated reperfusion for 4 h). The cells were randomized into six groups: control group (Con), Sirt3 knockdown group (Ad-sh-Sirt3), H/R group, Sirt3 knockdown + H/R group (Ad-sh-Sirt3+H/R), H/R+DHM group, and Sirt3 knockdown + H/R+DHM group (Ad-sh-Sirt3+H/R+DHM).

### 2.9. ATP, CS, and Mitochondrial DNA Content

For detecting the myocardial ATP content, an ATP bioluminescent assay kit (Sigma, USA) was performed [24]. Citrate synthase (CS) was measured using a citrate synthase activity assay kit (Sigma, USA). Mitochondria DNA content was measured as previously described [24].

### 2.10. MI Area

We used Evans Blue/TTC staining to detect the area of MI as previously described [25].

### 2.11. ROS and MnSOD

The ROS and MnSOD were detected as previously described [25].

### 2.12. Western Blot

The protein was separated by SDS-PAGE. The specific primary antibodies included Sirt3, TFAM, and NRF2 (1:1000, Cell Signaling, Danvers, MA, USA).

### 2.13. Statistics

Values are reported as means ± SEM. Comparison between groups was performed with ANOVA followed by Bonferroni correction for post hoc t-test. Values of P<0.05 were considered statistically significant.

## 3. Results

### 3.1. DHM Alleviated MI/R Injury

**Figure 1:**
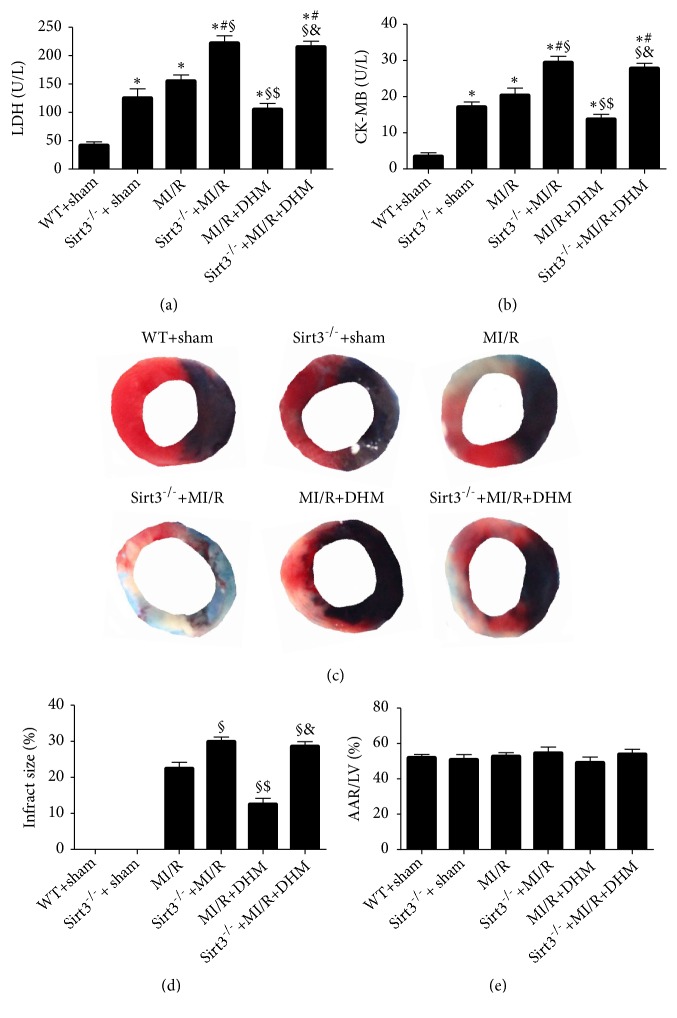
*DHM alleviated MI/R injury*. (a, b) Lactate dehydrogenase (LDH) and creatine kinase-MB (CK-MB). (c) The infarct area in each group. (d, e) Quantitative analysis of infarct area and AAR/LV. Mean ± SEM (n=12), ^*∗*^P<0.05 vs WT+sham group; ^#^P<0.05 vs Sirt3^−/−^ + sham group; ^§^P < 0.05 vs MI/R group; ^$^P<0.05 vs Sirt3^−/−^+MI/R group; ^&^P<0.05 vs MI/R +DHM group.

LDH and CK-MB levels were markedly decreased after DHM treatment in the mice after MI/R (Figures 1(a) and 1(b)). In addition, DHM slightly but insignificantly decreased their values in the Sirt3^−/−^ mice (Figures 1(a) and 1(b)). In the Sirt3^−/−^ hearts after MI/R, the infarct area was increased further compared with the area in the MI/R group. As shown in Figure 1(d), DHM decreased the infarct area in the mice after MI/R, but not in the Sirt3^−/−^ mice. No significant difference between the groups was observed in the ratio of the area at risk (AAR) to the left ventricular (LV) area (Figure 1(e)).

### 3.2. Treatment with DHM Improved Cardiac Function after MI/R

**Figure 2:**
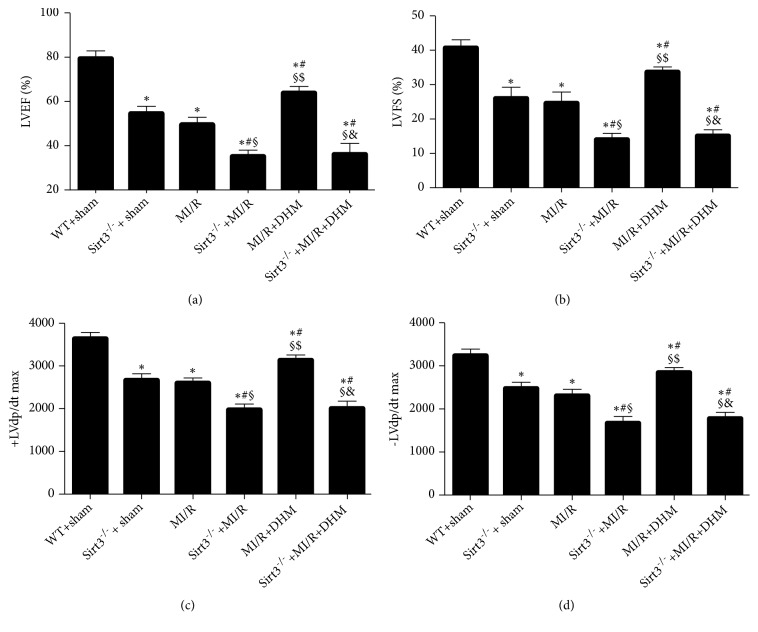
*DHM improved cardiac function after MI/R*. (a, b) LVEF, LVFS was measured by echocardiography. (c, d) The maximal ± LV dp / dt was obtained by hemodynamic evaluation. Mean ± SEM (n=12), ^*∗*^P<0.05 vs WT+sham group; ^#^P<0.05 vs Sirt3^−/−^ + sham group; ^§^P < 0.05 vs MI/R group; ^$^P<0.05 vs Sirt3^−/−^+MI/R group; ^&^P<0.05 vs MI/R +DHM group.

The LVEF, LVFS, and the ± LV dp/dt max were further decreased after MI/R in Sirt3^−/−^ mice (Figures 2(a)–2(d)). Supplementation with DHM significantly increased their values after MI/R, suggesting that DHM protected LV systolic function against MI/R (Figures 2(a)–2(d)). Specifically, we found that the LVEF and LVFS were not changed after DHM treatment in the mice without Sirt3 after MI/R (Figures 2(a) and 2(b)). Further, Sirt3 knockout resulted in the no effects of DHM on the maximal ± LV dp/dt (Figures 2(c) and 2(d)).

### 3.3. DHM Improved Mitochondrial Function after MI/R

**Figure 3:**
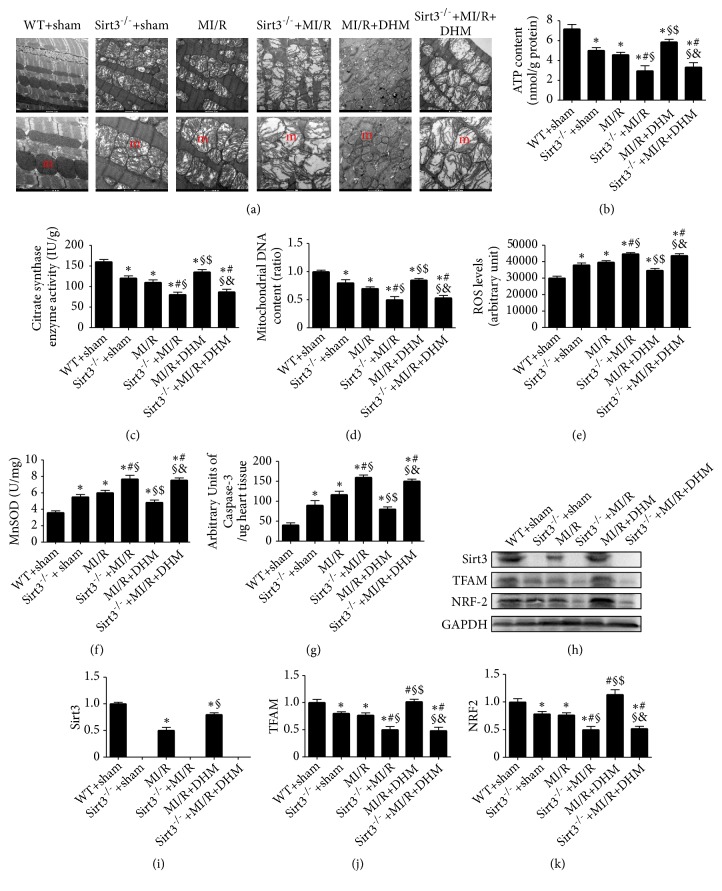
*DHM attenuated the mitochondrial dysfunction after MI/R*. (a) The morphology of mitochondria (m). (b, c) ATP content and citrate synthase (CS) activity. (d) Mitochondrial DNA content. (e) ROS levels. (f) Manganese superoxide dismutase (MnSOD). (g) Caspase-3 activity. (h-k) Representative bands shown for Sirt3, TFAM, and NRF-2 proteins are provided. Mean ± SEM (n=12), ^*∗*^P<0.05 vs WT+sham group; ^#^P<0.05 vs Sirt3^−/−^ + sham group; ^§^P < 0.05 vs MI/R group; ^$^P<0.05 vs Sirt3^−/−^+MI/R group; ^&^P<0.05 vs MI/R +DHM group.

In the Sirt3^−/−^ hearts and the MI/R hearts, the swelling, breakage, and disarrangement were observed in some mitochondria (Figure 3(a)). In Sirt3^−/−^ hearts, however, the vacuoles were observed in some mitochondria after MI/R, suggesting that the mitochondrial injury was aggravated. DHM inhibited the mitochondrial injury, as evident by the normal cristae in most mitochondria and less swollen mitochondria (Figure 3(a)). The mitochondrial ultrastructure disorder was not reduced after supplementation with DHM in Sirt3^−/−^ hearts after MI/R (Figure 3(a)). All the above data indicate that MI/R induced the mitochondrial dysfunction. DHM reduced the mitochondrial structure disorder after MI/R and removing Sirt3 abolished this effect of DHM (Figure 3(a)).

As mitochondria play a critical role in cardiac function after MI/R, we next assessed the mitochondrial function. As illustrated in Figures 3(b)–3(g), mitochondrial function was examined in difference groups. We found that MI/R induced the mitochondria dysfunction, as evident by lower ATP content, mitochondrial DNA content, and CS activity (Figures 3(b)–3(d)). Their values in the mitochondria were markedly increased in the MI/R+ DHM group (Figures 3(b)–3(d)). Together, these results indicate that DHM alleviated mitochondrial damage after MI/R. Interestingly, DHM could not alleviate these aberrant changes and had no effects on the mitochondrial DNA content, ATP content, or CS activity in the Sirt3^−/−^ mice after MI/R (Figures 3(b)–3(d)). ROS levels, mitochondrial MnSOD activity, and caspase-3 activity were reduced in the MI/R+DHM group. However, DHM had no effects on ROS levels, mitochondrial MnSOD activity, and caspase-3 activity in the Sirt3^−/−^ mice after MI/R ((Figures 3(e)–3(g)).

### 3.4. The Cardioprotection of DHM Was Directly Associated with the Sirt3/TFAM Pathway

In order to clarify the correlation of Sirt3, TFAM, and NRF2, we asked whether Sirt3 regulated TFAM and NRF2. Removing Sirt3 inhibited TFAM and NRF2 expression (Figures 3(h)–3(k)). In addition, Sirt3, TFAM, and NRF2 expression levels were somewhat reduced in MI/R mice (Figures 3(h)–3(k)). Our data revealed that Sirt3, TFAM, and NRF2 levels were increased after DHM treatment compared with levels in the nontreated hearts (Figures 3(h)–3(k)). DHM insignificantly increased TFAM and NRF2 levels in the mice without Sirt3 after MI/R (Figures 3(h)–3(k)). All of these observations lend support to the notion that Sirt3/TFAM signaling was associated with the MI/R and that DHM alleviated the MI/R injury by activating Sirt3/TFAM signaling.

### 3.5. DHM Alleviated Hypoxia/Reoxygenation Injury in Primary Cardiomyocytes

**Figure 4:**
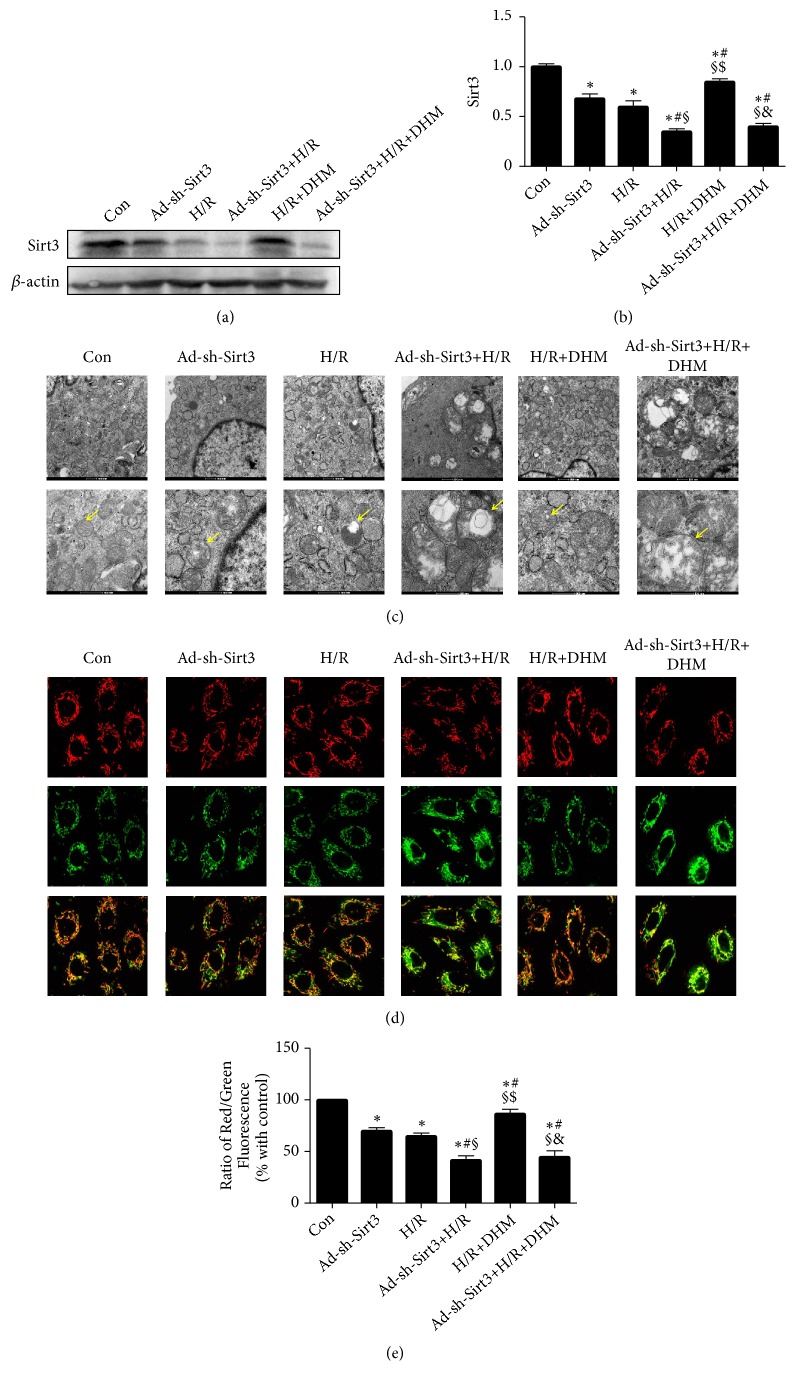
*DHM improved mitochondrial function after H/R*. (a, b) Representative blots of Sirt3. (c) The morphology of mitochondria (yellow arrow) in cardiomyocytes. (d) JC-1 aggregates label normal mitochondria with polarized inner mitochondrial membranes (Red). JC-1 monomers represent DYm dissipation (Green). (e) Quantification of the DYm (n=50 in each group). Scale bars= 20 um. Mean ± SEM, ^*∗*^P<0.05 vs Con; ^#^P<0.05 vs Ad-sh-Sirt3; ^§^P < 0.05 vs H/R; ^$^P<0.05 vs Ad-sh-Sirt3+H/R; ^&^P<0.05 vs H/R+DHM.

We showed that DHM significantly alleviated H/R-induced injury in cardiomyocytes, consistent with previous observations (Figure 4). Ad-sh-Sirt3 successfully reduced Sirt3 expression (Figures 4(a) and 4(b)). In addition, the Sirt3 expression level was somewhat decreased in cardiomyocytes after H/R injury. Sirt3 expression was increased in the DHM-treated cells, when compared with relative controls (Figures 4(a) and 4(b)).

### 3.6. DHM Improved Mitochondrial Function after H/R Injury

In the control group, the mitochondria were normal and regularly arranged. In the Ad-sh-Sirt3 or H/R group, the swelling and breakage were observed in some mitochondria (Figure 4(c)). In the Ad-sh-Sirt3 +H/R group, however, the mitochondrial injury was increased, which was evident from the vacuoles in some mitochondria. The mitochondrial injury was alleviated in the H/R+DHM group, as evident by the normal cristae in most mitochondria and less swollen mitochondria. DHM could not reduce the mitochondrial ultrastructure disorder in the Ad-sh-Sirt3 +H/R +DHM group (Figure 4(c)). Collectively, DHM reduced the mitochondrial ultrastructure disorder in the H/R+DHM group but not in the Ad-sh-Sirt3 +H/R +DHM group (Figure 4(c)).

To further confirm the role of mitochondria in cardiomyocytes after H/R, we detected the level of mitochondrial membrane potential. Our data indicated that DHM resulted in an increase in mitochondrial membrane potential in response to the H/R in cardiomyocytes (Figures 4(d) and 4(e)). Interestingly, DHM could not alleviate mitochondrial dysfunction in the Ad-sh-Sirt3 +H/R +DHM group (Figures 4(d) and 4(e)). These data demonstrated that DHM alleviated H/R injury in cardiomyocytes by upregulating Sirt3 expression.

## 4. Discussion

The most effective treatment to patients with ischemic heart disease is to restore coronary artery blood flow quickly [1, 2]. However, reperfusion itself can induce injury to the myocardium [3]. An exploration of the mechanisms regulating cardiac I/R injury could prevent and alleviate the heart impairment. In our study, DHM administration alleviated cardiac I/R injury.

Multiple studies indicated that DHM has many beneficial effects in diseases [6, 26]. We used the WT and Sirt3^−/−^ mice to construct a cardiac I/R model to explore the effects of DHM. To test cardiac function among different groups, we measured markers of cardiac function (LDH and CK-MB), cardiac function parameters such as LVEF and LVFS, and TTC staining to observe the infarction area. Cardiac dysfunction was observed in the MI/R model [3]. The injury was alleviated after DHM treatment. The treatment of hearts with DHM significantly decreased infarction size and improved cardiac function, but this effect was inhibited by Sirt3 knockout. This result indicates that DHM alleviates the MI/R injury through the upregulation of Sirt3. However, further studies are required to elucidate the precise mechanism by which DHM regulates Sirt3 activity during MI/R injury.

Mitochondria are important for heart contraction in producing the ATP to ensure energy supply [17, 27]. The normal mitochondria are essential for the pumping force of the heart [12]. Mitochondrial dysfunction makes an important part of the pathological process of MI/R [11, 25, 27]. Ischemia/reperfusion causes a large accumulation of myocardial ROS, suggesting that oxidative stress may contribute to cardiac dysfunction [28]. Excessive ROS induce cell damage and, ultimately, cell death [19]. Specifically, excessive ROS would induce mitochondrial membrane depolarization, electron transport chain deterioration, apoptotic pathway activation, and cardiomyocyte death [28–30]. In this study, DHM administration reduced MI/R injury-induced ROS generation, which may contribute to preventing mitochondrial dysfunction. However, in the Sirt3^−/−^ mice, DHM failed to downregulate the ROS level. These results indicated that DHM alleviated the oxidative stress in MI/R injury by activating Sirt3. To examine the mitochondrial function, we observed the mitochondrial morphology by TEM and detected the mitochondrial functional protein by Western blot. In our study, DHM increased ATP content, CS activity, and alleviated mitochondrial ultrastructure impairment after MI/R. More importantly, removing Sirt3 blocked the effects of DHM on mitochondrial biogenesis. We demonstrated that DHM protected against MI/R injury through Sirt3 activation.

Sirt3 has protective effects in many cardiovascular diseases [18]. Sirt3 in cardiomyocytes was inhibited after MI/R injury. Consistently, DHM increased the expression of mitochondrial function protein Sirt3, TFAM, and NRF-2 after MI/R. NRF-2 and TFAM, which are the mitochondrion-related genes, regulate mitochondrial biogenesis. Sirt3 induced NRF-2 gene expression, resulting in an increase in the oxidative phosphorylation-related protein expression, and thus regulates mitochondrial function [31]. Specifically, TFAM regulates the mtDNA copy number and maintains the stability of mtDNA [32]. DHM activates the Sirt3/TFAM pathway to protect against MI/R. A substantial loss of cardiomyocytes after MI/R induced contractile dysfunction [28, 30]. Moreover, DHM inhibited the cardiomyocytes apoptosis, as shown via caspase-3 activity. The antiapoptotic effects of DHM were abolished by removing Sirt3. Therefore, DHM may also inhibit the apoptosis through Sirt3 activation. In line with these observations, DHM decreased the infarction size, alleviated cardiac dysfunction, decreased cardiomyocyte oxidative stress and apoptosis, and improved mitochondrial function by activating Sirt3. Having demonstrated that the cardiac function is associated with the mitochondria biogenesis, and H/R induces the death of cardiomyocytes by damaging the mitochondria [25, 33], in the present study, DHM alleviated mitochondrial impairment in vitro study and this effect was abolished by Sirt3 knockdown. Furthermore, mitochondrial membrane potential was a marker of mitochondrial function, and it was decreased after H/R and increased after DHM treatment. However, the protective effects of DHM on the primary cardiomyocytes after H/R were abolished after downregulating Sirt3. These results indicated that DHM may protect mitochondrial function in primary cardiomyocytes against H/R injury by Sirt3 activation.

In conclusion, our current study provides convincing evidence that DHM decreases the infarction area and enhances cardiac function after MI/R. DHM may hold tremendous promise to treat or prevent heart diseases.

## Data Availability

The data used to support the findings of this study are included within the article.
